# Mathematical epidemiology: Past, present, and future

**DOI:** 10.1016/j.idm.2017.02.001

**Published:** 2017-02-04

**Authors:** Fred Brauer

**Affiliations:** University of British Columbia, Vancouver, BC, Canada

## Abstract

We give a brief outline of some of the important aspects of the development of mathematical epidemiology.

## 1 Introduction

Communicable diseases have always been an important part of human history. Since the beginning of recorded history there have been epidemics that have invaded populations, often causing many deaths before disappearing, possibly recurring years later, possibly diminishing in severity as populations develop some immunity. For example, the “Spanish” flu epidemic of 1918–19 caused more than 50,000,000 deaths worldwide, and there are annual influenza seasonal epidemics that cause up to 35,000 deaths worldwide.

The Black Deaths (probably bubonic plague) spread from Asia throughout Europe in several waves during the fourteenth century, beginning in 1346, and is estimated to have caused the death of as much as one-third of the population of Europe between 1346 and 1350. The disease recurred regularly in various parts of Europe for more than 300 years, notably as the Great Plague of London of 1665–1666. It then gradually withdrew from Europe.

There are also diseases that have become endemic (always present) in some populations and cause many deaths. This is especially common in developing countries with poor health care systems. Every year millions of people die of measles, respiratory infections, diarrhea, and other diseases that are easily treated and not considered dangerous in the Western world. Diseases such as malaria, typhus, cholera, schistosomiasis, and sleeping sickness are endemic in many parts of the world. The effects of high disease mortality on mean life span and of disease debilitation and mortality on the economy in afflicted countries are considerable. The World Health Organization has estimated that in 2011 there were 1,400,000 deaths due to tuberculosis, 1,200,000 deaths due to HIV/AIDS, and 627,000 deaths due to malaria (but other sources estimate the number of malaria deaths to have been more than 1,000,000). In 1980 there were 2,600,000 deaths due to measles, but the number of measles deaths in 2011 was reduced to 160,000, primarily because of the development of a measles vaccine.

The goal of epidemiologists is first to understand the causes of a disease, then to predict its course, and finally to develop ways of controlling it, including comparisons of different possible approaches. The first step is obtaining and analyzing observed data.

## 2 Some history

The study of infectious disease data began with the work of John Graunt (1620–1674) in his 1662 book “Natural and Political Observations made upon the Bills of Mortality”. The Bills of Mortality were weekly records of numbers and causes of death in London parishes. The records, beginning in 1592 and kept continuously from 1603 on, provided the data that Graunt used. He analyzed the various causes of death and gave a method of estimating the comparative risks of dying from various diseases, giving the first approach to a theory of competing risks.

What is usually described as the first model in mathematical epidemiology is the work of Daniel Bernoulli (1700–1782) on inoculation against smallpox. In the eighteenth century smallpox was endemic. Variolation, essentially inoculation with a mild strain, was introduced as a way to produce lifelong immunity against smallpox, but with a small risk of infection and death. There was heated debate about variolation, and Bernoulli was led to study the question of whether variolation was beneficial. His approach was to calculate the increase in life expectancy if smallpox could be eliminated as a cause of death. His approach to the question of competing risks led to publication of a brief outline in 1760 (Bernoulli, 1760) followed in 1766 by a more complete exposition (Bernoulli, 1766, pp. 1–45). His work received a mainly favorable reception but has become better known in the actuarial literature than in the epidemiological literature. However, more recently his approach has been generalized (Dietz & Heesterbeek, 2002).

Another valuable contribution to the understanding of infectious diseases even before there was knowledge about the disease transmission process was the knowledge obtained by study of the temporal and spatial pattern of cholera cases in the 1855 epidemic in London by John Snow, who was able to pinpoint the Broad Street water pump as the source of the infection (Johnson, 2006, Snow, 1855). In 1873, William Budd was able to achieve a similar understanding of the spread of typhoid (Budd, 1873). In 1840, William Farr studied statistical returns with the goal of discovering the laws that underlie the rise and fall of epidemics (Farr, 1840, pp. 91–98).

## 3 The beginnings of compartmental models

In order to describe a mathematical model for the spread of a communicable disease, it is necessary to make some assumption about the means of spreading infection. The modern view is that diseases are spread by contact through a virus or bacterium. The idea of invisible living creatures as agents of disease goes back at least to the writings of Aristotle (384 BCE–322 BCE). The existence of microorganisms was demonstrated by van Leeuwenhoek (1632–1723) with the aid of the first microscopes. The first expression of the germ theory of disease by Jacob Henle (1809–1885) came in 1840 and was developed by Robert Koch (1843–1910), Joseph Lister (1827–1912), and Louis Pasteur (1822–1875) in the late nineteenth and early twentieth centuries.

In 1906 W.H. Hamer proposed that the spread of infection should depend on the number of susceptible individuals and the number of infective individuals (Hamer, 1906). He suggested a mass action law for the rate of new infections, and this idea has been basic in compartmental models since that time. It is worth noting that the foundations of the entire approach to epidemiology based on compartmental models were laid, not by mathematicians, but by public health physicians such as Sir R.A. Ross, W.H. Hamer, A.G. McKendrick, and W.O. Kermack between 1900 and 1935.

A particularly instructive example is the work of Ross on malaria. Dr. Ross was awarded the second Nobel Prize in Medicine in 1902 for his demonstration of the dynamics of the transmission of malaria between mosquitoes and humans.

It was generally believed that so long as mosquitoes were present in a population malaria could not be eliminated. However, Ross gave a simple compartmental model (Ross, 1911) including mosquitoes and humans which showed that reduction of the mosquito population below a critical level would be sufficient. This was the first introduction of the concept of the basic reproduction number, which has been a central idea in mathematical epidemiology since that time. Field trials supported this conclusion and led to sometimes brilliant successes in malaria control.

The basic compartmental models to describe the transmission of communicable diseases are contained in a sequence of three papers by Kermack and McKendrick, 1927, Kermack and McKendrick, 1932, Kermack and McKendrick, 1933. The first of these papers described epidemic models.

The original formulation of the Kermack-McKendrick epidemic model given in Kermack and McKendrick (1927) was

(1) $$\begin{array}{l} v\left(t\right)=-x^{\prime }\left(t\right)\\ x^{\prime }\left(t\right)=-x\left(t\right)\left[\int_{0}^{t}A\left(s\right)v\left(t-s\right)ds+A\left(t\right)y_{0}\right]\\ z^{\prime }\left(t\right)=\int_{0}^{t}C\left(s\right)v\left(t-s\right)ds+C\left(t\right)y_{0}\\ y\left(t\right)=\int_{0}^{t}B\left(s\right)v\left(t-s\right)ds+B\left(t\right)y_{0}\text{.} \end{array}$$

here, x(t) is the number of susceptibles, y(t) is the number of infectious individuals, and z(t) is the number of recovered individuals. Also φ(s) is the recovery rate when the age of infection is *s*, ψ(s) is the recovery rate at infection age *s*, and

$$B\left(s\right)=e^{-\int_{0}^{t}\psi \left(s\right)ds},\;A\left(s\right)=\varphi \left(s\right)B\left(s\right)\text{.}$$

It is assumed that there are no disease deaths, so that the total population size remains constant. Kermack and McKendrick did not bring the basic reproduction number into their analysis, but were able to derive a final size relation in the form

(2) $$\text{log}\frac{1-\frac{y_{0}}{N}}{1-p}=pN\int_{0}^{\infty }A\left(s\right)ds\text{,}$$

in which *N* is the total population size and *p* is the attack ratio

$$p=1-\frac{x_{\infty }}{N}\text{.}$$

If we define

$$S\left(t\right)=x\left(t\right),\;A\left(s\right)=B\left(s\right)=e^{-\gamma s},\;I\left(t\right)=\frac{N}{a}y\left(t\right)\text{,}$$

the model (3) can be reduced to the system

(3) $$\begin{array}{l} S^{\prime }=-aS\frac{I}{N}\\ I^{\prime }=aS\frac{I}{N}-\gamma I\text{,} \end{array}$$

which is the simple Kermack-McKendrick model. For many years, the model (3) was known as the Kermack-McKendrick epidemic model, overlooking the fact that it is a very special case of the actual Kermack-McKendrick model. The general model included dependence on age of infection, that is, the time since becoming infected, and can be used to provide a unified approach to compartmental epidemic models.

Various disease outbreaks, including the SARS epidemic of 2002–3, the concern about a possible H5N1 influenza epidemic in 2005, the H1N1 influenza pandemic of 2009, and the Ebola outbreak of 2014 have re-ignited interest in epidemic models, beginning with the reformulation of the Kermack-McKendrick model by Diekmann, Heesterbeek, & Metz (1995).

In the work of Ross and Kermack and McKendrick there is a threshold quantity, the basic reproduction number, which is now almost universally denoted by ${ℛ}_{0}$. Neither Ross nor Kermack and McKendrick identified this threshold quantity or gave it a name. It appears that the first person to name the threshold quantity explicitly was MacDonald (1957) in his work on malaria.

The explicit definition of the basic reproduction number is that it is the expected number of disease cases produced by a typical infected individual in a wholly susceptible population over the full course of the infectious period. In an epidemic situation, in which the time period is short enough to neglect demographic effects and all infected individuals recover with full immunity against re-infection, the threshold ${ℛ}_{0}=1$ is the dividing line between the infection dying out and the onset of an epidemic. In a situation that includes a flow of new susceptibles, either through demographic effects or recovery without full immunity against re-infection, the threshold ${ℛ}_{0}=1$ is the dividing line between approach to a disease-free equilibrium and approach to an endemic equilibrium, in which the disease is always present. For the simple Kermack-McKendrick model (3) it is easy to verify that

$${ℛ}_{0}=\frac{a}{\gamma }\text{.}$$

Since 1933 there has been a great deal of work on compartmental disease transmission models, with generalizations in many directions. We will return to this topic in a later section. In particular, it is assumed in Kermack and McKendrick, 1927, Kermack and McKendrick, 1932, Kermack and McKendrick, 1933 that stays in compartments are exponentially distributed. In the generalization to age of infection models (Section 5.1) we are able to assume arbitrary distributions of stay in a compartment.

## 4 Stochastic models

There are serious shortcomings in the simple Kermack-McKendrick model as a description of the beginning of a disease outbreak, and a very different kind of model is required. The simple Kermack-McKendrick compartmental epidemic model assumes that the sizes of the compartments are large enough that the mixing of members is homogeneous. However, at the beginning of a disease outbreak, there is a very small number of infective individuals and the transmission of infection is a stochastic event depending on the pattern of contacts between members of the population; a description should take this pattern into account.

The process to be described is known as a Galton-Watson process, and the result was first given in Galton, (1889, pp. 241–248) and Watson and Galton (1874). There was a gap in the convergence proof, and the first complete proof was given much later by Steffensen (Steffensen, 1930, Steffensen, 1931). The result is a standard theorem given in many sources on branching processes, for example (Harris, 1963), but did not appear in the epidemiological literature until later. To the best of the author's knowledge, the first description in an epidemiological reference is (Metz, 1978) and the first epidemiological book source is the book by Diekmann and Heesterbeek (2000) in 2000.

A stochastic branching process description of the beginning of a disease outbreak begins with the assumption that there is a network of contacts of individuals, which may be described by a graph with members of the population represented by vertices and with contacts between individuals represented by edges. The study of graphs originated with the abstract theory of Erdös and Rényi of the 1950's and 1960's (Erdös and Rényi, 1959, Erdös and Rényi, 1960, Erdös and Rényi, 1961), and has become important more recently in many areas, including social contacts, computer networks, and many other areas as well as the spread of communicable diseases. We will think of networks as bi-directional, with disease transmission possible in either direction along an edge.

An edge is a contact between vertices that can transmit infection. The number of edges of a graph at a vertex is called the *degree* of the vertex. The degree distribution of a graph is $\left\{p_{k}\right\}$, where $p_{k}$ is the fraction of vertices having degree *k*. The degree distribution is fundamental in the description of the spread of disease. We assume that all contacts between an infective and a susceptible transmit infection, but this assumption can be relaxed.

We think of a small number of infectives in a population of susceptibles large enough that in the initial stage we may neglect the decrease in the susceptible population. We assume that the infectives make contacts independently of one another and let $p_{k}$ denote the probability that the number of contacts by a randomly chosen individual is exactly *k*, with $\sum_{k=0}^{\infty }p_{k}=1$. In other words, $\left\{p_{k}\right\}$ is the degree distribution of the vertices of the graph corresponding to the population network.

We define the *generating function*

$$F_{0}\left(z\right)=\sum_{k=0}^{\infty }p_{k}z^{k}\text{.}$$

Since $\sum_{k=0}^{\infty }p_{k}=1$, this power series converges for 0≤z≤1, and may be differentiated term by term. Thus

$$p_{k}=\frac{F_{0}^{\left(k\right)}\left(0\right)}{k!},\;k=0,1,2,\cdots \text{.}$$

It is easy to verify that the generating function has the properties

$$F_{0}\left(0\right)=p_{0},\;F_{0}\left(1\right)=1,\;F_{0}^{\prime }\left(z\right)>0,\;F_{0}^{\prime \prime }\left(z\right)>0.$$

The mean number of secondary infections, which we have defined as ${ℛ}_{0}$, is $F_{0}^{\prime }\left(1\right)$.

We consider a disease outbreak that begins with a single infected individual (“patient zero”) who transmits infection to every individual to whom this individual is connected, that is, along every edge of the graph from the vertex corresponding to this individual. In other words, we assume that a disease outbreak begins when a single infective transmits infection to all of the people with whom he or she is in contact. Our development via branching processes is along the lines of that of Diekmann and Heesterbeek (2000). Another approach, using a contact network perspective taken in (Callaway et al., 2000, Newman, 2002, Newman et al., 2001) begins with an infected edge, corresponding to a disease outbreak started by an infective individual who passes the infection on to only one contact. This approach is the one taken more commonly in studies of epidemics on networks. It is somewhat more complicated and leads to somewhat different results, although the methods are quite similar.

It is possible to prove that the probability $z_{\infty }$ that the infection will die out and will not develop into a major epidemic is the limit as n→∞ of the solution of the difference equation

$$z_{n}=F_{0}\left(z_{n-1}\right),\;z_{0}=0.$$

Thus $z_{\infty }$ must be an equilibrium of this difference equation, that is, a solution of $z=F_{0}\left(z\right)$. In fact, $z_{\infty }$ must be the smallest root of $F_{0}\left(z\right)=z$ .If ${ℛ}_{0}<1$ the equation $F_{0}\left(z\right)=z$ has only one root, namely z=1. On the other hand, if ${ℛ}_{0}>1$ the equation $F_{0}\left(z\right)=z$ has a second root $z_{\infty }<1$.

To summarize this analysis, we see that if ${ℛ}_{0}<1$ the probability that the infection will die out is 1, while if ${ℛ}_{0}>1$ there is a positive probability $1-z_{\infty }$ that the infection will persist, and will lead to an epidemic. However, there is a positive probability $z_{\infty }$ that the infection will increase initially but will produce only a minor outbreak and will die out before triggering a major epidemic. This distinction between a minor outbreak and a major epidemic, and the result that if ${ℛ}_{0}>1$ there may be only a minor outbreak and not a major epidemic are aspects of stochastic models not reflected in deterministic models.

One possible approach to a realistic description of an epidemic would be to use a branching process model initially and then make a transition to a compartmental model when the epidemic has become established and there are enough infectives that mass action mixing in the population is a reasonable approximation. Another approach would be to continue to use a network model throughout the course of the epidemic (Miller, 2011, Miller and Volz, 2011, Volz, 2008). It is possible to formulate this model dynamically, and the limiting case of this dynamic model as the population size becomes very large is the same as the compartmental model.

A basic description of network models may be found in Pourbohloul and Miller (2008). The theoretical analysis of network models is a very active and rapidly developing field (Meyers, 2007, Meyers et al., 2005, Meyers et al., 2006). A new addition to the network epidemiology literature is (Kiss, Miller, & Simon, 2017). The network approach to disease modeling is a rapidly developing field of study, and there will undoubtedly be fundamental developments in our understanding of the modeling of disease transmission. Some useful references are (Meyers, 2007, Meyers et al., 2006, Newman, 2002, Newman et al., 2001, Strogatz, 2001). There has been a move to complicated network models for simulating epidemics (Ferguson et al., 2005, Gani et al., 2005, Longini and Halloran, 2005, Longini et al., 2004, Longini et al., 2005, Meyers, 2007). These assume knowledge of the mixing patterns of groups of members of the population and make predictions based on simulations of a stochastic model.

Deterministic models are not appropriate for small populations because the spread of infection is a random process. For this reason, stochastic models have an important role in disease transmission modeling. The most commonly used stochastic model is the chain binomial model of Reed and Frost, first described in lectures in 1928 by W.H. Frost but not published until much later (Abbey, 1952, Wilson and Burke, 1942). The Reed - Frost model was actually anticipated nearly forty years earlier by P.D. En'ko (En'ko, 1889). The work of En'ko was brought to public attention much later by Karl Dietz (Dietz, 1988). E.B. Wilson and M.H. Burke have given a description of Frost's 1928 lectures with a somewhat different derivation (Wilson & Burke, 1942). M. Greenwood gave a somewhat different chain binomial model in 1931 (Greenwood, 1931). The Reed-Frost model has been used widely as a basic stochastic model and many extensions have been formulated. The book (Daley & Gani, 1999) by D.J. Daley and J. Gani contains an account of some of the more recent extensions. Also, a stochastic analogue of the Kermack-McKendrick epidemic model has been described in Bartlett (1949).

## 5 Developments in compartmental models

In the mathematical modeling of disease transmission, as in most other areas of mathematical modeling, there is always a trade-off between simple, or strategic, models, which omit most details and are designed only to highlight general qualitative behavior, and detailed, or tactical, models, usually designed for specific situations including short-term quantitative predictions. Detailed models are generally difficult or impossible to solve analytically and hence their usefulness for theoretical purposes is limited, although their strategic value may be high.

For example, very simple models for epidemics predict that an epidemic will die out after some time, leaving a part of the population untouched by disease, and this is also true of models that include control measures. This qualitative principle is not by itself very helpful in suggesting what control measures would be most effective in a given situation, but it implies that a detailed model describing the situation as accurately as possible might be useful for public health professionals.

It is important to recognize that mathematical models to be used for making policy recommendations for management need quantitative results, and the models needed in a public health setting require a great deal of detail in order to describe the situation accurately. For example, if the problem is to recommend what age group or groups should be the focus of attention in coping with a disease outbreak, it is essential to use a model which separates the population into a sufficient number of age groups and recognizes the interaction between different age groups. The increased availability of high - speed computing in recent years has made use of such models possible.

Many of the early developments in the mathematical modeling of communicable diseases are due to public health physicians. The first known result in mathematical epidemiology is a defense of the practice of inoculation against smallpox in 1760 by Daniel Bernoulli, a member of a famous family of mathematicians (eight spread over three generations) who had been trained as a physician. The first contributions to modern mathematical epidemiology are due to P.D. En'ko between 1873 and 1894 (En'ko, 1889), and the foundations of the entire approach to epidemiology based on compartmental models were laid by public health physicians such as Sir R.A. Ross, W.H. Hamer, A.G. McKendrick, and W.O. Kermack between 1900 and 1935, along with important contributions from a statistical perspective by J. Brownlee.

The development of mathematical methods for the study of models for communicable diseases led to a divergence between the goals of mathematicians, who sought broad understanding, and public health professionals, who sought practical procedures for management of diseases. While mathematical modeling led to many fundamental ideas, such as the possibility of controlling smallpox by vaccination and the management of malaria by controlling the vector (mosquito) population, the practical implementation was always more difficult than the predictions of simple models. Fortunately, in recent years there have been determined efforts to encourage better communication, so that public health professionals can better understand the situations in which simple models may be useful and mathematicians can recognize that real - life public health questions are much more complicated than simple models.

In the study of compartmental disease transmission models the population under study is divided into compartments and assumptions are made about the nature and time rate of transfer from one compartment to another. For example, in an SIR model we divide the population being studied into three classes labeled *S*, *I*, and *R*. We let S(t) denote the number of individuals who are susceptible to the disease, that is, who are not (yet) infected at time *t*. I(t) denotes the number of infected individuals, assumed infectious and able to spread the disease by contact with susceptibles. R(t) denotes the number of individuals who have been infected and then removed from the possibility of being infected again or of spreading infection.

In many diseases infectives return to the susceptible class on recovery because the disease confers no immunity against reinfection. Such models are appropriate for most diseases transmitted by bacterial or helminth agents, and most sexually transmitted diseases (including gonorrhea, but not such diseases as AIDS from which there is no recovery). We use the terminology SIS to describe a disease with no immunity against re-infection, to indicate that the passage of individuals is from the susceptible class to the infective class and then back to the susceptible class.

We will use the terminology SIR to describe a disease which confers immunity against re-infection, to indicate that the passage of individuals is from the susceptible class *S* to the infective class *I* to the removed class *R*. Usually, diseases caused by a virus are of SIR type.

In addition to the basic distinction between diseases for which recovery confers immunity against reinfection and diseases for which recovered members are susceptible to reinfection, and the intermediate possibility of temporary immunity signified by a model of SIRS type, more complicated compartmental structure is possible. For example, there are SEIR and SEIS models, with an exposed period between being infected and becoming infective.

The rates of transfer between compartments are expressed mathematically as derivatives with respect to time of the sizes of the compartments. Initially, we assume that the duration of stay in each compartment is exponentially distributed and as a result models are formulated initially as differential equations. Models in which the rates of transfer depend on the sizes of compartments over the past as well as at the instant of transfer lead to more general types of functional equations, such as differential-difference equations or integral equations. One way in which models have expressed the idea of a reduction in contacts as an epidemic proceeds is to assume a contact rate of the form βSf(I) with a function f(I) that grows more slowly than linearly in *I*. Such an assumption, while not really a mechanistic model, may give better approximation than simple mass action contact to observed data.

The development and analysis of compartmental models has grown rapidly since the early models. Many of these developments are due to Hethcote, 1976, Hethcote, 1978, Hethcote, 1989, Hethcote, 1997, Hethcote, 2000b. We describe only a few of the important developments. While there are three basic compartmental disease transmission models, namely the SIS model, the SIR model without births and deaths, and the SIR model with births and deaths, each disease has its own properties which should be included in a model for this disease.

For influenza, there is a significant fraction of the population which is infected but asymptomatic, with lower infectivity than symptomatic individuals. There are seasonal outbreaks which may be the same strain as the previous year but modified by mutation of the strain, and there is some cross-immunity protecting individuals who were infected by a similar strain in a previous year. Also, influenza models may include the effect of a partially efficacious vaccination before an outbreak and antiviral treatment during an outbreak. Cholera may be transmitted both by direct contact and by contact with pathogen shed by infectives in a water supply.

In tuberculosis some infected individuals progress rapidly to the infectious stage while others progress much more slowly. Also, in tuberculosis individuals who fail to comply with treatment schedules may develop a drug-resistant strain. In HIV/AIDS, the infectivity of an individual depends very strongly on the time since infection. In malaria, immunity against infection is boosted by exposure to infection.

### 5.1 The infection age epidemic model

Various disease outbreaks, including the SARS epidemic of 2002–3, the concern about a possible H5N1 influenza epidemic in 2005, the H1N1 influenza pandemic of 2009, and the Ebola outbreak of 2014 have re-ignited interest in epidemic models, beginning with the reformulation of the Kermack-McKendrick model by Diekmann et al. (1995). The basic assumptions are that individuals in the population make an average of *a* contacts sufficient to transmit infection in unit time and that the total population size is a constant *N* (assuming no disease deaths). Then the rate of contacts made by a susceptible that produce a new infection is aφ(t)/N, where φ(t) is the total infectivity of infected individuals, the number of infective individuals multiplied by their average relative infectivity. The number of new infections at time (t−s) is $\left[-S^{\prime }\left(t-s\right)\right]$ and on average the infectivity of these new infections at time *t* is A(s). Thus the total infectivity at time *t* is

$$\varphi \left(t\right)=\int_{0}^{\infty }A\left(s\right)S^{\prime }\left(t-s\right)ds\text{.}$$

Then

(4) $$\begin{array}{l} S^{\prime }\left(t\right)=-\frac{a}{N}S\left(t\right)\varphi \left(t\right)\\ \varphi \left(t\right)=-\int_{0}^{\infty }A\left(s\right)S\text{'}\left(t-s\right)ds=\frac{a}{N}\int_{0}^{\infty }A\left(s\right)S\left(t-s\right)\varphi \left(t-s\right)ds\text{.} \end{array}$$

The model (4) is more general than the model (3) in two respects. It allows an arbitrary distribution of stays in each compartment. It also allows an arbitrary sequence of infective compartments, and thus includes models with treatment, or quarantine and isolation. Then

(5) $${ℛ}_{0}=a\int_{0}^{\infty }A\left(s\right)ds\text{,}$$

and there is a final size relation

$$\text{log}\frac{S_{0}}{S_{\infty }}={ℛ}_{0}\left[1-\frac{S_{\infty }}{N}\right]\text{.}$$

There have been many presentations of this final size relation in various contexts, including models with heterogeneity of mixing (Andreasen, 2011, Brauer, 2008a, Brauer, 2008b, Brauer and Watmough, 2009, Breda et al., 2012, Diekmann and Heesterbeek, 2000, Diekmann et al., 1995, Ma and Earn, 2006).

### 5.2 Endemic disease models

The analytic approaches to models for endemic diseases and epidemics are quite different. The analysis of a model for an endemic disease begins with the search for equilibria, which are constant solutions of the model. Usually there is a disease-free equilibrium and there are one or more endemic equilibria, with a positive number of infected individuals. The next step is to linearize about each equilibrium and determine its stability. Usually, if the basic reproduction number is less than 1, the only equilibrium is the disease-free equilibrium and this equilibrium is asymptotically stable. If the basic reproduction number is greater than 1, the usual situation is that the disease-free equilibrium is unstable and there is a unique endemic equilibrium which is asymptotically stable. This approach also covers diseases in which there is vertical transmission, which is direct transmission from mother to offspring at birth (Busenberg & Cooke, 1993).

However, more complicated behavior is possible. For example, if there are two strains of the disease being studied it is common to have regions in the parameter space in which there is an asymptotically stable equilibrium with only one of the strains present and a region in which there is an asymptotically stable equilibrium with both strains coexisting. Another possibility is that there is a unique endemic equilibrium but it is unstable. In this situation there is often a Hopf bifurcation and an asymptotically stable periodic orbit around the endemic equilibrium. An example of such behavior may be found in an SIRS model, with a temporary immunity period of fixed length following recovery (Hethcote, Stech, & van den Driessche, 1981). If there is a periodic orbit with large amplitude and a long period, data must be gathered over a sufficiently large time interval to give an accurate picture.

Another possible behavior is a backward bifurcation. As ${ℛ}_{0}$ increases through 1 there is an exchange of stability between the disease-free equilibrium, which is asymptotically stable for ${ℛ}_{0}<1$ and unstable for ${ℛ}_{0}>1$, and the endemic equilibrium which exists if ${ℛ}_{0}>1$. The usual transition is a forward, or transcritical, bifurcation at ${ℛ}_{0}=1$, with an asymptotically stable endemic equilibrium and an equilibrium infective population size depending continuously on ${ℛ}_{0}$.

The behavior at a bifurcation may be described graphically by the bifurcation curve, which is the graph of equilibrium infective population size *I* as a function of the basic reproductive number ${ℛ}_{0}$. It has been noted (Dushoff et al., 1998, Hadeler and Castillo-Chavez, 1995, Hadeler and van den Driessche, 1997, Kribs-Zaleta and Velasco-Hernandez, 2000) that in epidemic models with multiple groups and asymmetry between groups or multiple interaction mechanisms it is possible to have a very different bifurcation behavior at ${ℛ}_{0}=1$. There may be multiple positive endemic equilibria for values of ${ℛ}_{0}<1$ and a backward bifurcation at ${ℛ}_{0}=1$. The qualitative behavior of a system with a backward bifurcation differs from that of a system with a forward bifurcation and the nature of these changes has been described in Brauer (2004). Since these behavior differences are important in planning how to control a disease, it is important to determine whether a system can have a backward bifurcation.

### 5.3 Diseases transmitted by vectors

Many diseases are transmitted from human to human indirectly, through a vector. Vectors are living organisms that can transmit infectious diseases between humans. Many vectors are bloodsucking insects that ingest disease-producing microorganisms during blood meals from an infected (human) host, and then inject it into a new host during a subsequent blood meal. The best known vectors are mosquitoes for diseases including malaria, dengue fever, chikungunya, Zika virus, Rift Valley fever, yellow fever, Japanese encephalitis, lymphatic filariasis, and West Nile fever, but ticks (for Lyme disease and tularemia), bugs (for Chagas' disease), flies (for onchocerciasis), sandflies (for leishmaniasis), fleas (for plague, transmitted by fleas from rats to humans), and some freshwater snails (for schistosomiasis) are vectors for some diseases.

Every year there are more than a billion cases of vector-borne diseases and more than a million deaths. Vector-borne diseases account for over 17% of all infectious diseases worldwide. Malaria is the most deadly vector-borne diseases, causing an estimated 627,000 deaths in 2012. The most rapidly growing vector-borne disease is dengue, for which the number of cases has multiplied by 30 in the last 50 years. These diseases are found more commonly in tropical and sub-tropical regions where mosquitoes flourish and in places where access to safe drinking water and sanitation systems is uncertain.

Some vector-borne diseases, such as dengue, chikungunya, and West Nile virus are emerging in countries where they were unknown previously because of globalization of travel and trade and environmental challenges such as climate change. A troubling new development is the Zika virus, which has been known since 1952 but has developed a mutation in the South American outbreak of 2015 (Schuler-Faccini, 2016) which has produced very serious birth defects in babies born to infected mothers. In addition, the current Zika virus can be transmitted directly through sexual contact as well as through vectors.

Many of the important underlying ideas of mathematical epidemiology arose in the study of malaria begun by Sir. R.A. Ross (Ross, 1911). Malaria is one example of a disease with vector transmission, the infection being transmitted back and forth between vectors (mosquitoes) and hosts (humans). It kills hundreds of thousands of people annually, mostly children and mostly in poor countries in Africa. Among communicable diseases, only tuberculosis causes more deaths. Other vector diseases include West Nile virus, yellow fever, and dengue fever. Human diseases transmitted heterosexually may also be viewed as diseases transmitted by vectors, because males and females must be viewed as separate populations and disease is transmitted from one population to the other.

Vector transmitted diseases require models that include both vectors and hosts. For most diseases transmitted by vectors, the vectors are insects, with a much shorter life span than the hosts, who may be humans as for malaria or animals as for West Nile virus.

The compartmental structure of the disease may be different in host and vector species; for many diseases with insects as vectors an infected vector remains infected for life so that the disease may have an SI or SEI structure in the vectors and an SIR or SEIR structure in the hosts.

### 5.4 Heterogeneity of mixing

It has often been observed in epidemics that there is a small number of “superspreaders” who transmit infection to many other members of the population, while most infectives do not transmit infections at all or transmit infections to very few others. This suggests that homogeneous mixing at the beginning of an epidemic may not be a good approximation.

The SARS epidemic of 2002–3 spread much more slowly than would have been expected on the basis of the data on disease spread at the start of the epidemic. Early in the SARS epidemic of 2002–3 it was estimated that ${ℛ}_{0}$ had a value between 2.2 and 3.6. At the beginning of an epidemic, the exponential rate of growth of the number of infectives is approximately $\left({ℛ}_{0}-1\right)/\alpha$, where 1/α is the generation time of the epidemic, estimated to be approximately 10 days for SARS. This would have predicted at least 30,000 cases of SARS in China during the first four months of the epidemic. In fact, there were fewer than 800 cases reported in this time. An explanation for this discrepancy is that the estimates were based on transmission data in hospitals and crowded apartment complexes. It was observed that there was intense activity in some locations and very little in others. This suggests that the actual reproduction number (averaged over the whole population) was much lower, perhaps in the range 1.2−1.6, and that heterogeneous mixing was a very important aspect of the epidemic.

In disease transmission models not all members of the population make contacts at the same rate. In sexually transmitted diseases there is often a “core” group of very active members who are responsible for most of the disease cases, and control measures aimed at this core group have been very effective in control (Hethcote and Yorke, 1984). In epidemics there are often “super-spreaders”, who make many contacts and are instrumental in spreading disease and in general some members of the population make more contacts than others. To model heterogeneity in mixing we may assume that the population is divided into subgroups with different activity levels. Formulation of models requires some assumptions about the mixing between subgroups. There have been many studies of mixing patterns in real populations, for example (Blythe et al., 1991, Blythe et al., 1995, Busenberg et al., 1989, Mossong et al., 2008, Nold, 1980).

Age is one of the most important characteristics in the modeling of populations and infectious diseases. Individuals with different ages may have different reproduction and survival capacities. Diseases may have different infection rates and mortality rates for different age groups.

Individuals of different ages may also have different behaviors, and behavioral changes are crucial in control and prevention of many infectious diseases. Young individuals tend to be more active in interactions with or between populations, and in disease transmissions.

Sexually-transmitted diseases (STDs) are spread through partner interactions with pair-formations, and the pair-formations are clearly age-dependent in most cases. For example, most HIV cases occur in the group of young adults.

Childhood diseases, such as measles, chicken pox, and rubella, are spread mainly by contacts between children of similar ages. More than half of the deaths attributed to malaria are in children under 5 years of age due to their weaker immune systems. This suggests that in models for disease transmission in an age structured population it is necessary to allow the contact rates between two members of the population to depend on the ages of both members.

The development of age-structured models for disease transmission required development of the theory of age-structured populations. In fact, the first models for age-structured populations (McKendrick, 1926) were designed for the study of disease transmission in such populations.

### 5.5 The next generation method

In simple compartmental models, the basic reproduction number may be calculated by following the secondary cases caused by a single infective introduced into a population. However, if there are subpopulations with different susceptibility to infection it is necessary to follow the secondary infections in the subpopulations separately, and this approach will not yield the reproduction number. It is necessary to give a more general approach to the meaning of the reproduction number, and this is done through the *n*ext generation matrix (Diekmann and Heesterbeek, 2000, Diekmann et al., 1990, van den Driessche and Watmough, 2002). The underlying idea is that we must calculate the matrix whose (i,j) entry is the number of secondary infections caused in compartment *i* by an infected individual in compartment *j*.

In a compartmental disease transmission model we sort individuals into compartments based on a single, discrete state variable. A compartment is called a *d*isease compartment if the individuals therein are infected. Note that this use of the term disease is broader than the clinical definition and includes stages of infection such as exposed stages in which infected individuals are not necessarily infective. Suppose there are *n* disease compartments and *m* nondisease compartments, and let $x\in R^{n}$ and $y\in R^{m}$ be the subpopulations in each of these compartments. Further, we denote by $F_{i}$ the rate at which secondary infections increase the $i^{th}$ disease compartment and by $V_{i}$ the rate at which disease progression, death and recovery decrease the $i^{th}$ compartment. The compartmental model can then be written in the form

(6) $$\begin{array}{l} x_{i}^{\prime }=F_{i}\left(x,y\right)-V_{i}\left(x,y\right),\;i=1,\ldots ,n,\\ y_{j}^{\prime }=g_{j}\left(x,y\right),\;j=1,\ldots ,m\text{.} \end{array}$$

Note that the decomposition of the dynamics into F and V and the designation of compartments as infected or uninfected may not be unique; different decompositions correspond to different epidemiological interpretations of the model.

The derivation of the basic reproduction number is based on the linearization of the ODE model about a disease-free equilibrium. We assume

• $F_{i}\left(0,y\right)=0$ and $V_{i}\left(0,y\right)=0$ for all y≥0 and i=1,…,n.
• the disease-free system $y^{\prime }=g\left(0,y\right)$ has a unique equilibrium that is asymptotically stable, that is, all solutions with initial conditions of the form (0,y) approach a point $\left(0,y_{o}\right)$ as t→∞. We refer to this point as the disease-free equilibrium.

The first assumption says that all new infections are secondary infections arising from infected hosts; there is no immigration of individuals into the disease compartments. It ensures that the disease-free set, which consists of all points of the form (0,y), is invariant. That is, any solution with no infected individuals at some point in time will be free of infection for all time. The second assumption ensures that the disease-free equilibrium is also an equilibrium of the full system.

Next, we assume

• $F_{i}\left(x,y\right)\geq 0$ for all nonnegative *x* and *y* and i=1,…,n.
• $V_{i}\left(x,y\right)\leq 0$ whenever $x_{i}=0$, i=1,…,n.
• $\sum_{i=1}^{n}V_{i}\left(x,y\right)\geq 0$ for all nonnegative *x* and *y.*

The reasons for these assumptions are that the function F represents new infections and cannot be negative, each component, $V_{i}$, represents a net outflow from compartment *i* and must be negative (inflow only) whenever the compartment is empty, and the sum $\sum_{i=1}^{n}V_{i}\left(x,y\right)$ represents the total outflow from all infected compartments. Terms in the model leading to increases in $\sum_{i=1}^{n}x_{i}$ are assumed to represent secondary infections and therefore belong in F.

Suppose that a single infected person is introduced into a population originally free of disease. The initial ability of the disease to spread through the population is determined by an examination of the linearization of (6) about the disease-free equilibrium $\left(0,y_{o}\right)$. It is easy to see that the assumption $F_{i}\left(0,y\right)=0,V_{i}\left(0,y\right)=0$ implies

$$\frac{\partial F_{i}}{\partial y_{j}}\left(0,y_{o}\right)=\frac{\partial V_{i}}{\partial y_{j}}\left(0,y_{o}\right)=0$$

for every pair (i,j). This implies that the linearized equations for the disease compartments, *x*, are decoupled from the remaining equations and can be written as

(7) $$x^{\prime }=\left(F-V\right)x\text{,}$$

where *F* and *V* are the n×n matrices with entries

$$F=\frac{\partial F_{i}}{\partial x_{j}}\left(0,y_{o}\right)\;\text{and}\;V=\frac{\partial V_{i}}{\partial x_{j}}\left(0,y_{o}\right)\text{.}$$

Because of the assumption that the disease-free system y'=g(0,y) has a unique asymptotically stable equilibrium, the linear stability of the system (6) is completely determined by the linear stability of the matrix (F−V) in (7).

The number of secondary infections produced by a single infected individual can be expressed as the product of the expected duration of the infectious period and the rate at which secondary infections occur. It can be shown that the expected number of secondary infections produced by the index case is given by $FV^{-1}x_{0}.$ Following Diekmann and Heesterbeek (Diekmann & Heesterbeek, 2000), the matrix $K_{L}=FV^{-1}$ is referred to as the *n*ext generation matrix with large domain for the system at the disease-free equilibrium. The (i,j) entry of *K* is the expected number of secondary infections in compartment *i* produced by individuals initially in compartment *j*, assuming, of course, that the environment seen by the individual remains homogeneous for the duration of its infection.

The matrix, $K_{L}=FV^{-1}$ has a nonnegative eigenvalue, ${ℛ}_{0}=\rho \left(FV^{-1}\right)$, such that there are no other eigenvalues of *K* with modulus greater than ${ℛ}_{0}$ and there is a nonnegative eigenvector *ω* associated with ${ℛ}_{0}$ [ (Berman & Plemmons, 1994), Theorem1.3.2]. This eigenvector is in a sense the distribution of infected individuals corresponding to the number, ${ℛ}_{0}$, of secondary infections per generation. Thus, ${ℛ}_{0}$ and the associated eigenvector *ω* suitably define a “typical” infective and the basic reproduction number can be rigorously defined as the spectral radius of the matrix, $K_{L}$. The *s*pectral radius of a matrix $K_{L}$, denoted by $\rho \left(K_{L}\right)$, is the maximum of the moduli of the eigenvalues of $K_{L}$. If $K_{L}$ is irreducible, then ${ℛ}_{0}$ is a simple eigenvalue of $K_{L}$ and is strictly larger in modulus than all other eigenvalues of $K_{L}$. However, if $K_{L}$ is reducible, which is often the case for diseases with multiple strains, then $K_{L}$ may have several positive real eigenvectors corresponding to reproduction numbers for each competing strain of the disease.

It is possible to prove that ${ℛ}_{0}=\rho \left(FV^{-1}\right)<1$ if and only if all eigenvalues of (F−V) have negative real parts and that the disease-free equilibrium of (6) is locally asymptotically stable if ${ℛ}_{0}<1$, but unstable if ${ℛ}_{0}>1$.

In the simple Kermack-McKendrick epidemic model there are two parameters, the rate of new infections and the recovery rate. Often, the recovery rate for a particular disease is known. Typically in a disease outbreak there is a stochastic phase initially, followed by an exponential increase in the number of infectives, and it may be possible to estimate this initial exponential growth rate experimentally. If the initial exponential growth rate in an age of infection epidemic model with infectivity A(τ) at infection age *τ* is *ρ*, then

$${ℛ}_{0}=\frac{\int_{0}^{\infty }A\left(\tau \right)d\tau }{\int_{0}^{\infty }e^{-\rho \tau }A\left(\tau \right)d\tau }$$

(Diekmann et al., 1995, Wallinga and Lipsitch, 2007). This expression remains valid if the mixing in the model is not homogeneous.

## 6 Some current topics of interest

Frequently there have been outbreaks of new or recurring diseases, some of which have pointed to important epidemiological questions.

In their later work on disease transmission models (Kermack and McKendrick, 1932, Kermack and McKendrick, 1933), Kermack and McKendrick did not include age of infection, and age of infection models were neglected for many years. Age of infection reappeared in the study of HIV/AIDS, in which the infectivity of infected individuals is high for a brief period after becoming infected, then quite low for an extended period, possibly several years, before increasing rapidly with the onset of full-blown AIDS. Thus the age of infection described by Kermack and McKendrick for epidemics became very important in some endemic situations; see for example (Thieme and Castillo-Chavez, 1993, Thieme et al., 1989). Also, HIV/AIDS has pointed to the importance of immunological ideas in the analysis on the epidemiological level.

### 6.1 Spatial distributions

The SARS epidemic of 2002–3 emphasized the possibility of disease transmission over long distances through air travel, and this has led to metapopulation studies (Arino and van den Driessche, 2003a, Arino and van den Driessche, 2003b, Arino and van den Driessche, 2006, Arino et al., 2007). A metapopulation is a population of populations linked by travel between populations. For a disease model in a metapopulation, there would be a reproduction number for each patch as well as reproduction numbers taking account of travel between patches, which could be either temporary travel or permanent migration between patches.

A recent development in the study of spatial spread of diseases is the idea of *residence time* (Bichara et al., 2015, Castillo-Chavez et al., 2016). A perspective, which may be more appropriate in some situations, would be to describe patches with residents who spend a fraction of their time in different patches. For example, the spread of an infectious disease from one village to another through people who visit other patches may be a realistic description. Another interpretation could be to assume that individuals spend some of their time in environments more likely to allow disease transmission.

Thus we would consider two patches, with total resident population sizes $N_{1}$ and $N_{2}$ respectively, each population being divided into susceptibles, infectives, and removed members. $S_{i}$ and $I_{i}$ denote the number of susceptibles and infectives respectively who are residents in Patch *i*, regardless of the patch in which they are present. We consider an SIR epidemic model in two patches with different contact rates and with short term travel between the two patches. The total population resident in each patch is constant. We follow a Lagrangian perspective, that is, we keep track of each individual's place of residence at all times (Bichara et al., 2015, Castillo-Chavez et al., 2016). This is in contrast to a Eulerian perspective, which describes migration between patches.

Residents of Patch *i* spend a fraction $p_{ij}$ of their time in Patch *j*, with

$$p_{11}+p_{12}=1,\;p_{21}+p_{22}=1.$$

The contact rate in Patch *i* is $\beta_{i}$.

Each of the $p_{11}S_{1}$ susceptibles from Group 1 present in Patch 1 can be infected by infectives from Group 1 and from Group 2 present in Patch 1. Similarly, each of the $p_{12}S_{1}$ susceptibles present in Patch 2 can be infected by infectives from Group 1 and from Group 2 present in Patch 2. The density of infected individuals in Patch 1 at time t who can infect only individuals currently in Patch 1 at time t, that is, the *effective* infective proportion in Patch 1 is given by

$$p_{11}\frac{I_{1}\left(t\right)}{N_{1}}+p_{21}\frac{I_{2}\left(t\right)}{N_{2}}\text{.}$$

Thus the rate of new infections of members of Patch 1 in Patch 1 is

$$\beta_{1}p_{11}S_{1}\left(p_{11}\frac{I_{1}\left(t\right)}{N_{1}}+p_{21}\frac{I_{2}\left(t\right)}{N_{2}}\right)\text{.}$$

The rate of new infections of members of Patch 1 in Patch 2 is

$$\beta_{2}p_{12}S_{1}\left(p_{12}\frac{I_{1}\left(t\right)}{N_{1}}+p_{22}\frac{I_{2}\left(t\right)}{N_{2}}\right)\text{.}$$

There is a corresponding calculation for the rate of new infections of members of Patch 2 in each patch. From these calculations, we see that a model for an SIR infection is given by

$$S_{1}^{\prime }=-\beta_{1}p_{11}S_{1}\left[p_{11}\frac{I_{1}}{N_{1}}+p_{21}\frac{I_{2}}{N_{2}}\right]-\beta_{2}p_{12}S_{1}\left[p_{12}\frac{I_{1}}{N_{1}}+p_{22}\frac{I_{2}}{N_{2}}\right]$$

(8) $$I_{1}^{\prime }=\beta_{1}p_{11}S_{1}\left[p_{11}\frac{I_{1}}{N_{1}}+p_{21}\frac{I_{2}}{N_{2}}\right]+\beta_{2}p_{12}S_{1}\left[p_{12}\frac{I_{1}}{N_{1}}+p_{22}\frac{I_{2}}{N_{2}}\right]-\gamma I_{1}$$

$$S_{2}^{\prime }=-\beta_{1}p_{21}S_{2}\left[p_{11}\frac{I_{1}}{N_{1}}+p_{21}\frac{I_{2}}{N_{2}}\right]-\beta_{2}p_{22}S_{2}\left[p_{12}\frac{I_{1}}{N_{1}}+p_{22}\frac{I_{2}}{N_{2}}\right]$$

$$I_{2}^{\prime }=\beta_{1}p_{21}S_{2}\left[p_{11}\frac{I_{1}}{N_{1}}+p_{21}\frac{I_{2}}{N_{2}}\right]+\beta_{2}p_{22}S_{2}\left[p_{12}\frac{I_{1}}{N_{1}}+p_{22}\frac{I_{2}}{N_{2}}\right]-\gamma I_{2}$$

In this structure, actual travel between patches is not described explicitly, and this makes the analysis less complicated. Calculation of the reproduction number and the final size relations is possible.

Another aspect of the study of the spread of diseases is the spatial spread of diseases through diffusion. This has been examined in considerable detail in Rass and Ratcliffe (2003), with particular emphasis on epidemic waves.

### 6.2 Heterogeneity of mixing, cross immunity, coinfection

The SARS epidemic also pointed to the importance of heterogeneous mixing, especially nosocomial (in-hospital) transmission (Hsieh et al., 2014, Webb et al., 2004). During the SARS epidemic, where no treatment was available, the main management approach was isolation of diagnosed infectives and quarantine of suspected infectives. Quarantine was decided by tracing of contacts of infectives. In fact, very few quarantined individuals developed symptoms of SARS, and improvements in contact tracing are important for epidemic control.

In the models that we have been discussing, only one disease or disease strain has been involved. However, there are many situations in which more than one strain of a disease is present, and there may be cross-immunity between different strains (Andreasen, 2003, Andreasen et al., 1997). This may lead to models in which there is a disease-free equilibrium, equilibria in which only one strain persists, and an equilibrium in which two strains coexist.

Another possible situation is coinfection of more than one disease requiring more elaborate models. This is a real possibility with HIV and tuberculosis (Kirschner, 1999, Naresh and Tripathi, 2005, Porco et al., 2001, Raimundo et al., 2003, Schulzer et al., 1994, West and Thompson, 1997).

### 6.3 Drug resistance

The risk of development of resistance to drugs used in disease treatment has become a serious concern. In short-term disease outbreaks, antiviral treatment is one of the methods used to treat illness and also to decrease the basic reproduction number ${ℛ}_{0}$ and thus to lessen the number of cases of disease. However, many infectious pathogens can evolve and generate successor strains that confer drug-resistance (Domingo and Holland, 1997). The evolution of resistance is generally associated with impaired transmission fitness compared to the sensitive strains of the infectious pathogen (Moghadas, 2011). In the absence of treatment, resistant strains may be competitively disadvantaged compared to the sensitive strains and may go extinct. However, treatment prevents the growth and spread of sensitive strains, and therefore induces a selective pressure that favors the resistant strain to replicate and restore its fitness to a level suitable for successful transmission (Andersson & Levin, 1999). This phenomenon has been observed in several infectious diseases, in particular for management of influenza infection using antiviral drugs (Rimmelzwaan et al., 2005).

Previous models of influenza epidemics and pandemics have investigated strategies for antiviral treatment in order to reduce the epidemic final size (the total number of infections throughout the epidemic), while preventing wide-spread drug-resistance in the population (Hansen and Day, 2011, Lipsitch et al., 2007, Moghadas, 2008, Moghadas et al., 2008, Moghadas et al., 2009). Through computer simulations, these studies have shown that, when resistance is highly transmissible, there may be situations in which increasing the treatment rate may do more harm than good by causing a larger number of resistant cases than the decrease in cases produced by treatment of sensitive infections. A recent epidemic model (Xiao, Brauer, & Moghadas, 2016) has exhibited such behavior and suggested that there may be an optimal treatment rate for minimizing the final size (Lipsitch et al., 2007, Moghadas, 2008, Moghadas et al., 2008).

In diseases such as tuberculosis which operate on a very long time scale, the same problems arise but the modeling scenario is quite different. It is necessary to include demographic effects such as births and natural deaths in a model. This means that there may be an endemic equilibrium, and that the disease is always present in the population. Instead of studying the final size of an epidemic to measure the severity of a disease outbreak, it is more appropriate to consider the degree of prevalence of the disease in the population as a measure of severity. For diseases such as tuberculosis, in which there are additional aspects such as reinfection, there may be additional difficulties caused by the possibility of backward bifurcations. The importance of understanding the dynamics of tuberculosis treatment suggests that this is a topic that should be pursued.

### 6.4 Slower than exponential growth

It has been standard practice in analyzing disease outbreaks to formulate a dynamical system as a deterministic compartmental model, then to use observed early outbreak data to fit parameters to the model, and finally to analyze the dynamical system to predict the course of the disease outbreak and to compare the effects of different management strategies. In general, such models predict an initial stochastic stage (while the number of infectious individuals is small), followed by a period of exponential growth. Measurement of this early exponential growth rate is an essential step in estimating contact rate parameters for the model. A thorough description of the analysis of compartmental models may be found in Hethcote (2000a).

However, instances have been noted where the growth rate of an epidemic is clearly slower than exponential. For example, the initial apparently exponential spread of the 2013–2015 Ebola epidemic in West Africa can be viewed as a composition of locally asynchronous outbreaks at local level displaying sub-exponential growth patterns during several generations (Chowell, Viboud, Hyman, & Simonsen, 2015). Specifically, if I(t) is the number of infectious individuals at time *t*, a graph of logI(t) against *t* is a straight line if the growth rate is exponential, and for some disease outbreaks this has not been true. One of the earliest examples (Colgate, Stanley, Hyman, Layne, & Qualls, 1989) concerns the growth of HIV/AIDS in the United States, and a possible explanation might be the mixture of short-term and long-term contacts. This could be a factor in other diseases where there are repeated contacts in family groups and less frequent contacts outside the home.

It has been pointed out (Chowell et al., 2015, Chowell et al., 2016a, Chowell et al., 2016b, Viboud et al., 2016) that a so-called general growth model of the form

$$C^{\prime }\left(t\right)=rC{\left(t\right)}^{p}\text{,}$$

where C(t) is the number of disease cases occurring up to time *t*, and p,0≤p≤1, is a “deceleration of growth’ parameter, has exponential solutions if p=1 but solutions with polynomial growth if 0<p<1. This is not and does not claim to be a mechanistic epidemic model, but it has proved to be remarkably successful for fitting epidemic growth and predicting the future course of an epidemic. For example, it has provided much better estimates of epidemic final size than the exponential growth assumption for the Ebola epidemic of 2014. Such phenomenological models are particularly likely to be suitable in situations where it is difficult to construct a mechanistic approach because of multiple transmission routes, interactions of spatial influences, or other aspects of uncertainty.

A variety of epidemiological situations in which slower than exponential epidemic growth might be possible have been described. Ultimately, the challenge for epidemiological modeling would be to determine which of these situations allow slower than exponential growth by deriving and analyzing mechanistic models to describe each of these situations. This is an important new direction for epidemic modeling. Some suggestions include metapopulation models with spatial structure including cross-coupling and mobility, clustering in spatial structure, dynamic contacts, agent-based models with differences in infectivity and susceptibility of individuals, and reactive behavioral changes early in a disease outbreak. It may well turn out that slower than exponential growth may be ruled out in some cases but is possible in others. For example, heterogeneity of mixing in a single location can be modelled by an autonomous dynamical system and the linearization theory of dynamical systems at an equilibrium shows that early epidemic growth for such a system is always exponential. On the other hand, metapopulation models may well allow many varieties of behaviors.

Another direction that would be well worth further exploration would be contact rates decreasing in time because of individual behavioral changes in response to a disease outbreak. A contact rate which is a decreasing function of time can certainly lead to early epidemic growth slower than exponential. A step in this direction has been initiated in a discrete model (Fisman, Hauck, Tuite, & Greer, 2013) that has been applied to an Ebola model in (Fisman, Khoo, & Tuite, 2014).

An important broader question is the matter of what information influences the behavior of people during a disease outbreak, and how to include this in a model. A recent book (Manfredi and d'Onofrio, 2013) describes some studies in this direction.
